# Robustness of Bio-Inspired Visual Systems for Collision Prediction in Critical Robot Traffic

**DOI:** 10.3389/frobt.2021.529872

**Published:** 2021-08-06

**Authors:** Qinbing Fu, Xuelong Sun, Tian Liu, Cheng Hu, Shigang Yue

**Affiliations:** ^1^ Machine Life and Intelligence Research Centre, School of Mechanical and Electrical Engineering, Guangzhou University, Guangzhou, China; ^2^ School of Computer Science, University of Lincoln, Lincoln, United Kingdom

**Keywords:** bio-inspired computation, collision prediction, robust visual systems, LGMDs, micro-robot, critical robot traffic

## Abstract

Collision prevention sets a major research and development obstacle for intelligent robots and vehicles. This paper investigates the robustness of two state-of-the-art neural network models inspired by the locust’s LGMD-1 and LGMD-2 visual pathways as fast and low-energy collision alert systems in critical scenarios. Although both the neural circuits have been studied and modelled intensively, their capability and robustness against real-time critical traffic scenarios where real-physical crashes will happen have never been systematically investigated due to difficulty and high price in replicating risky traffic with many crash occurrences. To close this gap, we apply a recently published robotic platform to test the LGMDs inspired visual systems in physical implementation of critical traffic scenarios at low cost and high flexibility. The proposed visual systems are applied as the only collision sensing modality in each micro-mobile robot to conduct avoidance by abrupt braking. The simulated traffic resembles on-road sections including the intersection and highway scenes wherein the roadmaps are rendered by coloured, artificial pheromones upon a wide LCD screen acting as the ground of an arena. The robots with light sensors at bottom can recognise the lanes and signals, tightly follow paths. The emphasis herein is laid on corroborating the robustness of LGMDs neural systems model in different dynamic robot scenes to timely alert potential crashes. This study well complements previous experimentation on such bio-inspired computations for collision prediction in more critical physical scenarios, and for the first time demonstrates the robustness of LGMDs inspired visual systems in critical traffic towards a reliable collision alert system under constrained computation power. This paper also exhibits a novel, tractable, and affordable robotic approach to evaluate online visual systems in dynamic scenes.

## 1 Introduction

The World Health Organisation (WHO) reported that every year, approximately 1.35 millions people worldwide die on road traffic with an increase of 0.11 million over only 5 years ago (WHO, 2018). Collision prevention is an old, but active topic in research communities since it is still obstructing the development of intelligent robots and vehicles. For examples, the internet of vehicles (IoV) systems and technologies are confronting huge challenges from traffic accidents where the emergent strategies from deep learning (Chang et al., 2019) and cloud communication (Zhou et al., 2020) are improving the IoV’s reliability. The unmanned aerial vehicles (UAVs) industries are also reflecting on how to enhance the capability of obstacle detection and avoidance especially when flying through unstructured, dynamic scenes (Albaker and Rahim, 2009). On-road crashes usually occur randomly which are difficult to predict and trace. The typical accident-prone places include intersections, road junctions and highways, etc., where collision prevention is difficult to tackle (Mukhtar et al., 2015).

Therefore, a critically important task is the development of collision avoidance systems with ultimate reliability, which nevertheless is faced with huge challenges (Zehang Sun et al., 2006; Sivaraman and Trivedi, 2013; Mukhtar et al., 2015). The cutting-edge techniques for collision prediction include global positioning system (GPS), active (Radar, Laser, Lidar), and passive (acoustic and optical) sensor strategies, as well as combinations of these with sensor-based algorithms (Zehang Sun et al., 2006; Mukhtar et al., 2015). More specifically, the GPS has been used for predicting real time trajectory of vehicles for collision risk estimation (Ammoun and Nashashibi, 2009). The vision-based techniques have been implemented in passive sensors, i.e., the different kinds of cameras (Sun et al., 2004; Mukhtar et al., 2015). Compared to the active sensors like the Radar, the main advantages of visual ones are lower price and wider coverage of detection range up to 360°, which can provide much richer description about the vehicle’s surroundings including motion analysis (Sabzevari and Scaramuzza, 2016). Compared to the IoV and GPS techniques, the optical methods are not restricted by surrounding infrastructures (Mukhtar et al., 2015). However, the visual approaches bring about pronounced challenges upon fast implementation in real time, and accurate extraction of colliding features from the dynamic visual world mixed with many distractors. A reliable, real-time, energy-efficient collision alert visual system has not yet been demonstrated so far.

Fortunately, nature has been providing us with a lot of inspirations to construct collision sensing visual systems. Robust and efficient collision prediction system is ubiquitous amongst the vast majority of sighted animals. As a source of inspiration, the insects’ dynamic vision systems have been explored as powerful paradigms for collision detection and avoidance with numerous applications in machine vision, as reviewed in (Franceschini, 2014; Serres and Ruffier, 2017; Fu et al., 2018a, Fu et al., 2019b). As a prominent example, locusts can migrate for a long distance in dense swarms containing hundreds to thousands of individuals free of collision (Kennedy, 1951). In the locust’s visual pathways, two lobula giant movement detectors (LGMDs), i.e., the LGMD-1 and the LGMD-2, have been gradually identified and recognised to play crucial roles of collision perception, both of which respond most strongly to approaching objects signalling a direct collision course over other categories of visual movements like translating, receding, etc. (O’Shea and Williams, 1974; O’Shea and Rowell, 1976; Rind and Bramwell, 1996; Simmons and Rind, 1997; Rind and Simmons, 1999; Gabbiani et al., 2002, Gabbiani et al., 2004; Fotowat and Gabbiani, 2011; Rind et al., 2016; Yakubowski et al., 2016). More precisely, the LGMD releases bursts of energy whenever a locust is on a collision course with its cohorts or a predator bird. These energy by neural pulses leads to evasive actions like jumping from the ground while standing, or sliding while flying (Simmons et al., 2010). Surprisingly, the whole process from visual processing to behavioural response takes less than 50 milliseconds (Simmons et al., 2010; Sztarker and Rind, 2014). Therefore, building artificial visual systems that possess the similar robustness and timeliness like the locust’s LGMDs can undoubtedly benefit collision avoidance systems in intelligent robots and vehicles.

Learning from the locust’s LGMDs visual pathways and circuits, there have been many modelling studies to investigate either the LGMD-1 or the LGMD-2 against various visual scenes including online, wheeled mobile robots (Blanchard et al., 2000; Yue and Rind, 2005; Badia et al., 2010; Fu et al., 2016; Fu et al., 2017; Fu et al., 2018b; Isakhani et al., 2018; Fu et al., 2019b), walking robot (Cizek et al., 2017; Cizek and Faigl, 2019), UAVs (Salt et al., 2017, Salt et al., 2019; Zhao et al., 2019), and off-line car driving scenarios, e.g. (Keil et al., 2004; Stafford et al., 2007; Krejan and Trost, 2011; Hartbauer, 2017; Fu et al., 2019a, Fu et al., 2020a). These studies have demonstrated the effectiveness of LGMDs models as quick visual collision detectors for machine vision applications. However, due to high risk and price to replicate extremely dangerous traffic scenarios including many severe crashes, a vacancy is still there to investigate the capability and robustness of LGMDs models for addressing collision challenges from more critical scenarios where many physical collisions will happen. Regarding off-line testing approach, there is currently no comprehensive database covering real crash situations from vehicle-mounted cameras.

To fill these gaps, the recently published robotic platform named “*ColCOS*Φ” (Sun et al., 2019) can enrich the existing experimenting “toolbox” in the context. The platform can be used to physically implement different multi-robot traffic mimicking real world on-road collision challenges for investigating the proposed visual systems in a practical, affordable manner. More precisely, an artificial pheromones module herein works effectively to optically render different roadmaps involving lanes and signals upon a wide LCD screen acting as the ground of an arena for robots which can pick up these cues with light sensors at bottom. Accordingly, the robots can tightly follow the paths in navigation. Moreover, to focus on investigating the proposed LGMDs inspired visual systems, we apply very basic switch control to separate the states between normal navigation (going forward) and collision avoidance (abrupt braking). Here the more complex motion strategies resembling either the locust’s evasive behaviours or the ground vehicle’s actions are outside the scope of this study.

Therefore, the main contributions of this paper can be summarised as follows:

• This research corroborates the robustness of LGMDs neuronal systems model to timely alert potential crashes in dynamic multi-robot scenes. To sharpen up the acuity of LGMDs inspired visual systems in collision sensing, an original hybrid LGMD-1 and LGMD-2 neural networks model is proposed with non-linear mapping from network outputs to alert firing rate, which works effectively.
• This research complements previous experimentation on the proposed bio-inspired computation approach to collision prediction in more critical, real-physical scenarios.
• This paper exhibits an innovative, tractable, and affordable robotic approach to evaluate online visual systems in different dynamic scenes.

The rest of this article is structured as follows. Section 2 elaborates on the biological background, the formulation of proposed model, and the robotic platform. Section 3 presents the experimental settings on different types of robot traffic systems. Section 4 elucidates the results with analysis. Section 5 discusses on limitations and future works. Section 6 concludes this article.

## 2 Methods and Materials

### 2.1 Biological Inspiration

**FIGURE 1:**
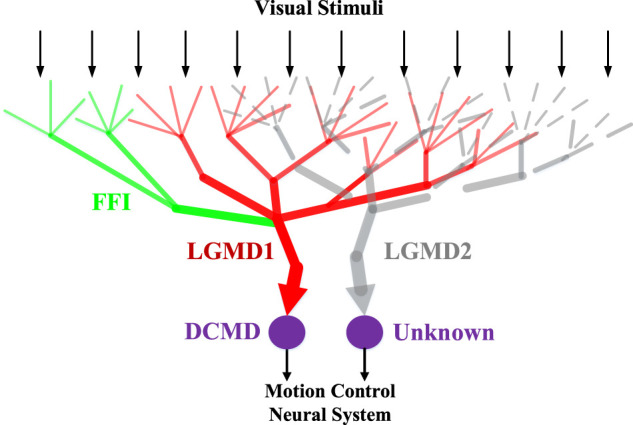
Schematic illustration of the LGMD-1 and the LGMD-2 neuromorphology. Visual stimuli are received by the pre-synaptic dendrite structures of both neurons. The feed-forward inhibition (FFI) pathway connects the LGMD-1. The DCMD (descending contra-lateral movement detector) is a one-to-one post-synaptic target neuron to the LGMD-1 conveying spikes to motion control neural system. The post-synaptic partner neuron to the LGMD-2 yet remains unknown.

Within this subsection, we firstly introduce the bio-inspiration, i.e., characterisation of the locust LGMD-1 and LGMD-2 visual neurons for the proposed computational modelling and experimenting. Figure 1 illustrates the schematic neural structures: the two neurons are physically close to each other. In general, they have been discovered amongst a group of LGMDs in the locust’s visual brain, a place called “lobula area” (O’Shea and Williams, 1974; O’Shea and Rowell, 1976). The LGMD-1 was first identified as a movement detector, and gradually recognised as a looming (approaching) objects detector, which responds most strongly to direct, rapid approaching objects rather than any other kinds of movements (Rind and Bramwell, 1996). In the same place, the LGMD-2 was also identified as a looming objects detector but with different selectivity to the LGMD-1, that is, the LGMD-2 is only sensitive to darker objects that approach against a relatively brighter background; whilst the LGMD-1 can detect either lighter or darker approaching objects (Simmons and Rind, 1997; Sztarker and Rind, 2014).

More precisely, both the looming perception visual neurons show increasing firing rates before the moving object reaching a particular angular size in the field of vision. They are rigorously inhibited at the end of objects approaching, the start of objects receding, and during transient luminance change over a large field of view. Against translating movements at constant speed, they are only activated very briefly. Most importantly, through our previous modelling and bio-robotic research (Fu et al., 2016; Fu et al., 2018b; Fu et al., 2019b), we have found that though with different selectivity, both the neurons could respond strongly to movements with increasing motion intensity, such as fast approaching and accelerating translating objects. Accordingly, all these specific characteristics make the LGMD-1 and LGMD-2 unique neuronal systems to model for addressing collision challenges for intelligent robots and vehicles.

### 2.2 Model Description

The collision selectivity, which indicates the neuron should respond most strongly to approaching objects over other kinds of movements, is a key feature to be realised in such looming perception neural network models separating their functionality from other categories of motion sensitive neural models (Fu et al., 2019b). Through hundreds of millions of years evolution, the locust’s LGMDs have been tuned with perfect collision selectivity, whereas the current computational models are not. In this regard, we have proposed a few effective methods to implement the different selectivity between the two LGMDs, and to sharpen up the selectivity via the modelling of bio-plausible ON/OFF channels (Fu et al., 2016; Fu et al., 2017; Fu et al., 2019b), and neural mechanisms like spike frequency adaptation (Fu et al., 2017; Fu et al., 2018b), and adaptive inhibition (Fu et al., 2019a; Fu et al., 2020b). However, the collision selectivity of current models still needs to be enhanced especially in complex and dynamic visual scenes.

**FIGURE 2:**
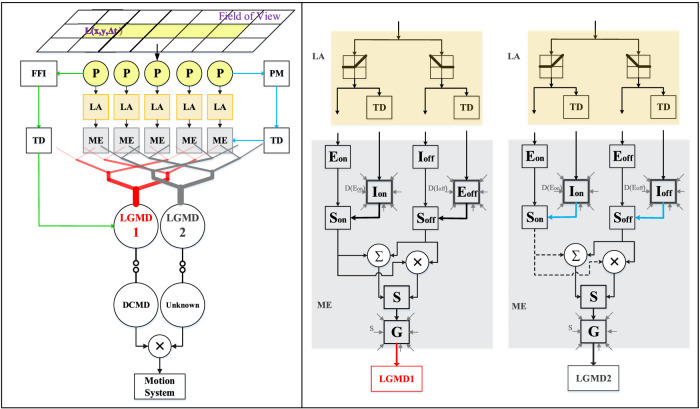
Schematic illustration of the proposed feed-forward collision prediction visual neural networks. There are three layers pre-synaptic to the two neurons, the photoreceptor (P), lamina (LA) and medulla (ME) layers. The pre-synaptic neural networks of LGMD-1 and LGMD-2 share the same visual processing in the first two, P and LA layers. The processing yet differs in the third ME layer for the purpose of separating their different selectivity. The ME layer consists of ON/OFF channels wherein the ON channels are rigorously suppressed in the LGMD-2’s circuit (dashed lines). The delayed information is formed by convolving surrounding non-delayed signals in space. The FFI is an individual inhibition pathway to merely the LGMD-1. The PM is a mediation pathway to the medulla layer of the LGMD-2. The two LGMDs pool their pre-synaptic signals respectively to generate spikes that are passed to their post-synaptic neurons. Notably, the non-linearly mapped, hybrid firing rate is the network output deciding the corresponding collision avoidance response.

Moreover, through previous studies, we have found the LGMD-2’s specific selectivity can complement the LGMD-1’s when detecting darker approaching objects, since the LGMD-1 is shortly activated by the recession of darker object whereas the LGMD-2 is not (Fu et al., 2019b). This well matches the situations faced by ground mobile robots and vehicles since most on-road objects are relatively darker than their backgrounds, particularly in daytime navigation. An interesting question thus arises that whether the two neuronal systems can coordinate in sculpting the dark looming selectivity. Accordingly, building upon the two state-of-the-art LGMDs neural network models (Fu et al., 2018b; Fu et al., 2019b), we propose a hybrid visual neural networks model combining the functionality of both the LGMD-1 and the LGMD-2, and investigate the robustness in dynamic visual scene. Compared to related networks, the proposed network features a non-linear unit for the product of spikes elicited by the LGMD-1 and the LGMD-2 neurons to generate the hybrid firing rate. This works effectively to sharpen up the selectivity to darker approaching objects over other motion patterns like recession. Consequently, a potential collision is detected only when both the LGMDs neurons are highly activated in the context. Figure 2 illustrates the schematic structure of the hybrid visual neural networks. The nomenclature is given in Table 1.

**TABLE 1:** Nomenclature in the visual neural networks.

Acronym and full-name	
LGMD	Lobula giant movement detector
DCMD	Descending contra-lateral movement detector
FFI	Feed-forward inhibition
P	Photoreceptor
LA/ME	Lamina/medulla neuron
PM	Photoreceptor mediation
TD	Time delay unit
E/I	Excitation/inhibition
S/G	Summation/grouping

#### 2.2.1 Photoreceptors Layer

As shown in Figure 2, the LGMD-1 and the LGMD-2 possess the same visual processing in the first two pre-synaptic layers. The first layer is composed of photoreceptors arranged in a matrix sensing time-varying, single-channel luminance (grey-scale in our case). The photoreceptors compute temporal derivative of every pixel to get motion information. Let $L\left(x,y,t\right)\in R^{3}$ denote the input image streams, where *x*, *y*, and *t* are spatial and temporal positions, respectively. The current motion can be retrieved by

$$P\left(x,y,t\right)=L\left(x,y,t\right)-L\left(x,y,t-1\right)+\overset{n_{p}}{\underset{i=1}{\sum }}a_{i}P\left(x,y,t-i\right),\;\text{where}\;a_{i}={\left(1+e^{i}\right)}^{-1}.$$ (1)

The motion persistence is constituted by *n*_*p*_ frames.

In addition, the P-layer also indicates the whole-field luminance change with respect to time. This indicator is applied as the FFI in the LGMD-1 neural network, which can be obtained by averaging the overall luminance change at time *t*. That is,

$$F\left(t\right)=\overset{R}{\underset{x=1}{\sum }}\overset{C}{\underset{y=1}{\sum }}|P\left(x,y,t\right)|\cdot {\left(C\cdot R\right)}^{-1},$$ (2)

where *C* and *R* denote columns and rows of the visual field in pixels. In addition to that, the FFI goes through a time delay unit (see TD in Figure 2), defined as

$$\hat{F}\left(t\right)=\alpha_{1}F\left(t\right)+\left(1-\alpha_{1}\right)F\left(t-1\right),\;\alpha_{1}=\tau_{i}/\left(\tau_{f}+\tau_{i}\right).$$ (3)

*τ*_*f*_ stands for a time constant and *τ*_*i*_ is the time interval between consecutive frames of digital signals, both in milliseconds. Notably, here the FFI can directly shut down the LGMD-1 neuron if it overshoots *T*_*ffi*_, i.e., a predefined threshold.

While modelling the LGMD-2, we propose a temporal tuning mechanism, the PM in Figure 2, to adjust local inhibitions in the medulla layer of the LGMD-2. The computations of PM conform to Eqs. 2, 3 which are not restated here.

#### 2.2.2 Lamina Layer

Motion information inevitably induces luminance increment or decrement over time. As shown in Figure 2, the second lamina layer separates the relayed signals into parallel ON and OFF channels, at each node. More precisely, the luminance increment flows into the ON channel, whilst the decrement streams to the OFF channel with a sign-inverting operation. Both the channels retain positive inputs. That is,

$$\begin{array}{cc} & P_{on}\left(x,y,t\right)={\left[P\left(x,y,t\right)\right]}^{+}+\alpha_{2}P_{on}\left(x,y,t-1\right),\\ & P_{o\mathrm{ff}}\left(x,y,t\right)=-{\left[P\left(x,y,t\right)\right]}^{-}+\alpha_{2}P_{o\mathrm{ff}}\left(x,y,t-1\right). \end{array}$$ (4)

[*x*]^+^ and [*x*]^−^ denote max (0, *x*) and min (*x*, 0). In addition, a small fraction (*α*_2_) of previous signal is allowed to pass through.

#### 2.2.3 Medulla Layer

The medulla layer is the place where different collision selectivity between LGMD-1 and LGMD-2 is shaped. The visual processing thus differs in this layer. First, in the LGMD-1’s medulla, the delayed information varies in different polarity pathways. More precisely, in the ON channels, the local inhibition (*I*_*on1*_) is formed by convolving surrounding delayed excitations (*E*_*on1*_). The whole spatiotemporal computation can be defined as the following:

$$E_{on1}\left(x,y,t\right)\;\;=\;\;P_{on}\left(x,y,t\right),$$ (5)

$${\hat{E}}_{on1}\left(x,y,t\right)\;\;=\;\;\alpha_{3}E_{on1}\left(x,y,t\right)+\left(1-\alpha_{3}\right)E_{on1}\left(x,y,t-1\right),\;\alpha_{3}=\tau_{i}/\left(\tau_{1}+\tau_{i}\right),$$ (6)

$$I_{on1}\left(x,y,t\right)=\overset{r}{\underset{i=-r}{\sum }}\overset{r}{\underset{j=-r}{\sum }}{\hat{E}}_{on1}\left(x+i,y+j,t\right)W_{1}\left(i+r,j+r\right).$$ (7)

*τ*_1_ stands for the latency of excitatory signal. *r* indicates the radius of convolving area [*W*_1_] denotes the convolution kernel in the LGMD-1 that meets the following matrix:

$$W_{1}=\left[\begin{array}{ccc} 1/8 & 1/4 & 1/8\\ 1/4 & 0 & 1/4\\ 1/8 & 1/4 & 1/8 \end{array}\right]$$ (8)

In the LGMD-1’s OFF channels, the delay is nevertheless put forth on the inhibitory signal; the excitation is thus formed by convolving delayed lateral inhibitions. That is,

$$I_{o\mathrm{ff}1}\left(x,y,t\right)=P_{o\mathrm{ff}}\left(x,y,t\right),$$ (9)

$${\hat{I}}_{o\mathrm{ff}1}\left(x,y,t\right)\;\;=\;\;\alpha_{4}I_{o\mathrm{ff}1}\left(x,y,t\right)\;\;+\;\;\left(1-\alpha_{4}\right)I_{o\mathrm{ff}1}\left(x,y,t-1\right),\;\alpha_{4}\;\;=\;\;\tau_{i}/\left(\tau_{2}+\tau_{i}\right),$$ (10)

$$E_{o\mathrm{ff}1}\left(x,y,t\right)=\overset{r}{\underset{i=-r}{\sum }}\overset{r}{\underset{j=-r}{\sum }}{\hat{I}}_{o\mathrm{ff}1}\left(x+i,y+j,t\right)\cdot W_{2}\left(i+r,j+r\right).$$ (11)

Here the convolution kernel [*W*_2_] is set equally to *W*_1_ in Eq. 8.

Second, in the LGMD-2’s medulla, much stronger local inhibitions are put forth in all the ON channels forming a biased-ON pathway in order to achieve its specific selectivity to only darker objects (see dashed lines in Figure 2). More specifically, the generation of local excitation (*E*_*on2*_) and inhibition (*I*_*on2*_) in the LGMD-2’s ON channels conforms to the LGMD-1 yet with a different latency *τ*_3_. To implement the ‘bias’, the convolution kernel matrix [*W*_3_] is increased with self-inhibition as

$$W_{3}=\left[\begin{array}{ccc} 1/4 & 1/2 & 1/4\\ 1/2 & 2 & 1/2\\ 1/4 & 1/2 & 1/4 \end{array}\right]$$ (12)

In the LGMD-2’s OFF pathway, the neural computation is defined as

$$E_{o\mathrm{ff}2}\left(x,y,t\right)=P_{o\mathrm{ff}}\left(x,y,t\right),$$ (13)

$${\hat{E}}_{o\mathrm{ff}2}\left(x,y,t\right)=\alpha_{5}E_{o\mathrm{ff}2}\left(x,y,t\right)+\left(1-\alpha_{5}\right)E_{o\mathrm{ff}2}\left(x,y,t-1\right),\;\alpha_{5}=\tau_{i}/\left(\tau_{4}+\tau_{i}\right),$$ (14)

$$I_{o\mathrm{ff}2}\left(x,y,t\right)=\overset{r}{\underset{i=-r}{\sum }}\overset{r}{\underset{j=-r}{\sum }}{\hat{E}}_{o\mathrm{ff}2}\left(x+i,y+j,t\right)W_{4}\left(i+r,j+r\right).$$ (15)

[*W*_4_] fits the following matrix:

$$W_{4}=\left[\begin{array}{ccc} 1/8 & 1/4 & 1/8\\ 1/4 & 1 & 1/4\\ 1/8 & 1/4 & 1/8 \end{array}\right]$$ (16)

As illustrated in Figure 2, following the generation of local ON/OFF excitation and inhibition, there are local summation units in either the medulla layer. For the LGMD-1, the calculation is as the following:

$$\begin{array}{cc} S_{on1}\left(x,y,t\right)= & {\left[E_{on1}\left(x,y,t\right)-w_{1}\cdot I_{on1}\left(x,y,t\right)\right]}^{+},\\ S_{o\mathrm{ff}1}\left(x,y,t\right)= & {\left[E_{o\mathrm{ff}1}\left(x,y,t\right)-w_{2}\cdot I_{o\mathrm{ff}1}\left(x,y,t\right)\right]}^{+}. \end{array}$$ (17)

*w*_1_ and *w*_2_ are the local biases. Note that only the positive S unit signals will pass through to the subsequent circuit. Compared to the LGMD-1, the two local biases are time varying, adjusted by the PM pathway in the LGMD-2. That is,

$$w_{3}\left(t\right)=\text{max}\left(1,\frac{PM\left(t\right)}{T_{\mathrm{ffi}}}\right),\;w_{4}\left(t\right)=\text{max}\left(0.5,\frac{PM\left(t\right)}{T_{\mathrm{ffi}}}\right),$$ (18)

$$\begin{array}{cc} S_{on2}\left(x,y,t\right)= & {\left[E_{on2}\left(x,y,t\right)-w_{3}\left(t\right)\cdot I_{on2}\left(x,y,t\right)\right]}^{+},\\ S_{o\mathrm{ff}2}\left(x,y,t\right)= & {\left[E_{o\mathrm{ff}2}\left(x,y,t\right)-w_{4}\left(t\right)\cdot I_{o\mathrm{ff}2}\left(x,y,t\right)\right]}^{+}. \end{array}$$ (19)

In both the LGMDs, the polarity summation cells interact with each other in a supra-linear manner as

$$S\left(x,y,t\right)=S_{on}\left(x,y,t\right)+S_{o\mathrm{ff}}\left(x,y,t\right)+S_{on}\left(x,y,t\right)S_{o\mathrm{ff}}\left(x,y,t\right).$$ (20)

Cascaded the S unit, a grouping unit is introduced to reduce isolated motion and enhance the extraction of expanding edges of colliding objects in cluttered backgrounds. This is implemented with a passing coefficient matrix [*Ce*] determined by another convolving process with an equally weighted kernel [*W*_*g*_]. That is,

$$Ce\left(x,y,t\right)=\overset{r}{\underset{i=-r}{\sum }}\overset{r}{\underset{j=-r}{\sum }}S\left(x+i,y+j,t\right)\cdot W_{g}\left(i+r,j+r\right),$$ (21)

$$W_{g}=\frac{1}{9}\;\times \left[\begin{array}{ccc} 1 & 1 & 1\\ 1 & 1 & 1\\ 1 & 1 & 1 \end{array}\right]$$ (22)

$$G\left(x,y,t\right)=S\left(x,y,t\right)Ce\left(x,y,t\right)\omega {\left(t\right)}^{-1},\;\text{where}\;\omega \left(t\right)=\text{max}\left({\left[Ce\right]}_{t}\right)C_{\omega }^{-1}+\Delta_{C}.$$ (23)

*ω* is a scale parameter computed at every discrete time step. *C*_*ω*_ is a constant. Δ_*C*_ stands for a small real number. Here only the non-negative grouped signals can get through.

#### 2.2.4 LGMD-1 and LGMD-2 Neurons

After the signal processing of pre-synaptic neural networks, the LGMD-1 and LGMD-2 neurons integrate corresponding local excitations from the medulla layer to generate membrane potentials. Here we apply a sigmoid transformation as the neuron activation function. The whole process can be defined as

$$k\left(t\right)=\overset{R}{\underset{x=1}{\sum }}\overset{C}{\underset{y=1}{\sum }}G\left(x,y,t\right),\;K\left(t\right)={\left(1+e^{-k\left(t\right)\cdot {\left(C\cdot R\right)}^{-1}}\right)}^{-1},$$ (24)

Subsequently, a spike frequency adaptation mechanism is applied to sculpt the neural response to moving objects threatening collision. The computation is defined as follows:

$$\hat{K}\left(t\right)=\left\{\begin{array}{c} \alpha_{6}\left(\hat{K}\left(t-1\right)+K\left(t\right)-K\left(t-1\right)\right),\;\text{if}\;\left(K\left(t\right)-K\left(t-1\right)\right)\leq 0\\ \alpha_{6}K\left(t\right),\;\text{otherwise}, \end{array}\right.$$ (25)

$$\alpha_{6}=\tau_{s}/\left(\tau_{s}+\tau_{i}\right),$$ (26)

where *α*_6_ is a coefficient indicating the adaptation rate to visual movements calculated by the time constant *τ*_*s*_. Generally speaking, such a mechanism reduces neuronal firing rate to stimuli with constant or decreasing intensity, e.g., objects recede or translate at a constant speed; while it has little effect on accelerating motion with increasing intensity like the approaching.

#### 2.2.5 Hybrid Spiking

As the time interval between frames of digital signals is much longer than the reaction time of real visual neurons, we map the membrane potentials exponentially to spikes by an integer-valued function. That is,

$$S^{spike}\left(t\right)=\left[e^{\left(\alpha_{7}\cdot \left(\hat{K}\left(t\right)-T_{spi}\right)\right)}\right],$$ (27)

where *T*_*spi*_ denotes the spiking threshold and *α*_7_ is a scale coefficient affecting the firing rate, i.e., raising it will bring about more spikes within a specified time window.

As illustrated in Figure 2, the elicited spikes are conveyed to their post-synaptic target neurons. Differently from previous modelling on single neuron computation of either the LGMD-1 or the LGMD-2, we herein put forward a non-linear hybrid spiking mechanism aiming at improving the selectivity to darker objects that only threaten direct collision by suppressing the response to other categories of visual stimuli. As a result, the specific selectivity of LGMD-2 well complements the LGMD-1’s where the hybrid spiking frequency will be amplified merely when both neurons are activated. The computation is defined as

$$S_{h}^{spike}=\left\{\begin{array}{cc} & S_{2}^{spike},\;\text{if}\;\hat{F}\left(t\right)\geq T_{\mathrm{ffi}}\\ & S_{1}^{spike}\times S_{2}^{spike},\;\text{otherwise} \end{array}\right.$$ (28)

Finally, the detection of potential collision threat can be indicated by

$$Col\left(t\right)=\left\{\begin{array}{cc} & \text{True},\text{if}\left(\overset{t}{\underset{i=t-n_{t}}{\sum }}S_{h}^{spike}\left(i\right)\right)\times 1000/\left(n_{t}\cdot \tau_{i}\right)\geq T_{col}\\ & \text{False},\;\text{otherwise} \end{array}\right.$$ (29)

*n*_*t*_ denotes a short time window in frames. *T*_*col*_ stands for the collision warning threshold.

#### 2.2.6 Setting Network Parameters

Table 2 elucidates the parameters. In this study, we set up the parameters of neural networks depending on 1) prior knowledges from neuroscience (Rind and Bramwell, 1996; Simmons and Rind, 1997; Rind et al., 2016), 2) previous experience on modelling and experimenting of the LGMD-1 and the LGMD-2 neuron models (Fu et al., 2018b; Fu et al., 2019b), 3) considerations of fast implementation with optimisation as embedded vision systems for online visual processing (Hu et al., 2018). More concretely, the convolutional matrices *W*_1_, *W*_2_, *W*_3_, and *W*_4_ are not only based on previous biological and computational studies, but also optimised by “bitwise operation” on the embedded system. There is currently no feedback pathways and learning steps involved in the proposed hybrid neural networks. The parameters given in Table 2 have been systematically investigated in our previous bio-robotic studies with optimisation (Fu et al., 2018b; Fu et al., 2019b; Fu et al., 2017). In addition to that, the very limited computational resources in the micro-robot is restricted for online learning algorithms. Therefore, aligned with previous settings, the emphasis herein is laid on investigating the integration of both LGMDs inspired visual systems in robotic implementation of dynamic visual scenes.

**TABLE 2:** Setting network parameters.

Parameter	Description	Value
*n*_*p*_	Number of persistent frames	0
{*C*, *R*}	Columns, rows of the robot visual field	{99, 72}
*τ*_*i*_	Time interval in digital signal	1,000/30
*τ*_*f*_	Time constant in FFI-TD	90
*α*_2_	Small coefficient in LA	0.1
*r*	Radius of convolution kernel	1
*τ*_1_	Delay in LGMD-1 ON channels	30 in nearest cells, 60 diagonal
*τ*_2_	Delay in LGMD-1 OFF channels	30 in nearest cells, 60 diagonal
*τ*_3_	Delay in LGMD-2 ON channels	15 in centre, 30 nearest, 45 diagonal
*τ*_4_	Delay in LGMD-2 OFF channels	60 in centre, 120 nearest, 180 diagonal
{*w*_1_, *w*_2_}	Local inhibition biases in LGMD-1	{0.3, 0.6}
*C*_*ω*_	Constant in G units	4
Δ_*C*_	Small real number in G units	0.01
*τ*_*s*_	Time constant in spike frequency adaptation	500 ∼ 1,000
*T*_*ffi*_	Local threshold in activation of FFI	10
*α*_7_	Scale parameter in spiking mechanism	3 ∼ 6
*T*_*spi*_	Spiking threshold	0.7
*T*_*col*_	Collision warning threshold	40
*n*_*t*_	Time window to update spike frequency	10

### 2.3 Robotic Platform

**FIGURE 3:**
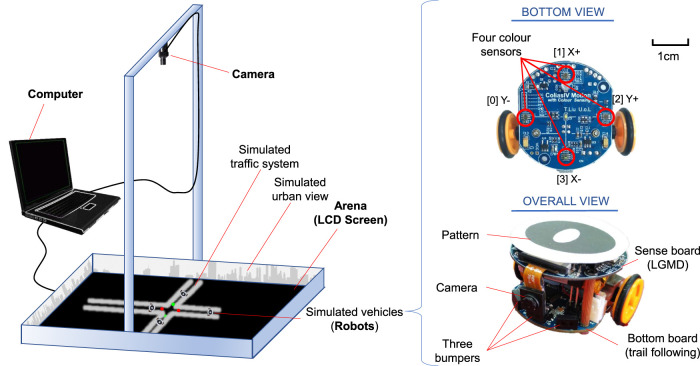
Overview of the robotic platform consisting of multiple-pheromone module and micro-mobile robots. The pheromone module is composed of a camera system connecting a computer and a TV arena. The micro-mobile robot comprises a visual sense board implementing the proposed visual systems, and a motion board for route following and emergency braking. Four colour sensors are marked in the bottom view of the robot used for sensing optically rendered pheromone cues displayed upon the LCD screen. The ID-pattern on top of robot is used to run a real time localisation system.

Within this subsection, we introduce the robotic platform, called *ColCOS*Φ (Sun et al., 2019), used to simulate different traffic scenarios in this research. As shown in Figure 3, the platform mainly consists of artificial multi-pheromone module, and autonomous micro-mobile robots.

#### 2.3.1 Artificial Pheromones Module

**FIGURE 4:**
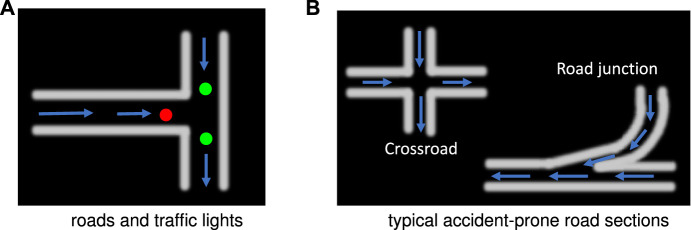
Using virtual pheromones to mimic roads and traffic lights: arrows indicate steering directions of robots. The roads are unidirectional.

Firstly, the multiple pheromones module was originally developed to conduct the swarm robotic experiments mimicking behaviours of social insects with interactions between multiple pheromones representing different biochemical substances (Sun et al., 2019). More specifically, as illustrated in Figure 3, the module consists of a camera system, a computer, and an arena with an LCD screen acting as the ground. The computer runs a pattern recognition algorithm in real time (Krajník et al., 2014), which is feasible to track and localise many ID-specific patterns with images at 1920 (pixels in width) × 1,080 (pixels in height) from the top-down facing camera, simultaneously, so as to record coordinates of robots with respect to time. In addition, the computer can render the virtual pheromone components, optically, represented by colour tracks or spots on the LCD screen indicating meaningful fields for mobile robots. Here the virtual pheromones are applied to render road maps and signals in the context. As shown in Figure 4, since the nature of pheromone field displayed on the LCD screen is a colour image, different traffic paradigms can be formed in which the roads are drawn by white tracks with boundaries, and the traffic lights and signals are embodied by green/red colour spots with appropriate size on the roads. Accordingly, different traffic sections like intersections, junctions, and even more complex road network can be established with scalability. Figure 4 shows some examples in our experiments. Together with periphery patterned walls (urban skyline), the arena is well constructed for our specific goal of simulating robotic traffic to test the proposed bio-inspired visual systems.

#### 2.3.2 Micro-Mobile Robot

As illustrated in Figure 3, the autonomous mobile robot used in this study is called *Colias-IV* (Hu et al., 2018), which includes mainly two components that provide different functions, namely the *Colias* Basic Unit (CBU), and the *Colias* Sensing Unit (CSU).

The CBU serves preliminary robot features such as motion, power management and some basic sensing like the bumper infra-red (IR) sensors in Figure 3. The more detailed configuration can be found in our recent paper (Hu et al., 2018), which is not reiterated here. Specifically for the proposed tasks, the CBU is assembled with four colour sensors with high sensitivity on its bottom (see Figure 3). When the robot is running in the arena, these sensors can pick up optical pheromone information on the LCD screen, and then the robot behaviours are adjusted accordingly.

**FIGURE 5:**
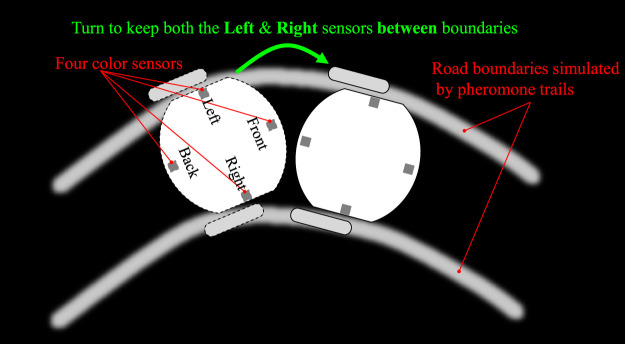
Robot route following strategy: keeping two-side colour sensors between road boundaries.

A key factor herein is tightly following the paths. We propose a control strategy that the two-side colour sensors are applied to bind the robot trajectory on the roads, as explained in Figure 5. Moreover, the front and rear light sensors play roles of recognising traffic signals including red (stop) and green (go) lights in the city traffic system, as well as accelerating and decelerating fields in the highway traffic system. More concrete control logic will be presented in the following Section 3.

The proposed LGMDs inspired visual systems are implemented for online visual processing. Here the CSU supports this where an ARM Cortex-M4F core micro controller is deployed as the main processor to handle intensive image processing. A monocular camera system with a low voltage CMOS image sensor OV7670 module is utilised in the CSU. With compact size, the camera is capable of operating up to 30 frames per second (fps) in VGA mode with output support for various colour formats. The horizontal viewing angle is approximately 70°. As a trade-off between processing efficiency and image quality, the resolution is configured at 72 × 99 pixels on 30 fps, with output format of 8-bit YUV422. Since the LGMDs only process grey-scale images, the camera setting fits it well, i.e., the proposed image format separates each pixel’s colour channels from the brightness channel; thus no additional colour transformation is required. More details of the CSU can be found in (Hu et al., 2018). Importantly, when assessing the proposed LGMDs inspired visual systems, the optical sensor is applied as the only collision detector.

Furthermore, the micro-robot can communicate with a host computer via a Bluetooth device connecting the CSU (Hu et al., 2018). Here we use it for retrieving the hybrid spiking frequency. With limited processing memory space and transmission ability, a current drawback of the robot is that it cannot send back real-time image views accompanied by motion.

## 3 Setting Experiments

Within this section, we introduce the experimental settings on multi-robot traffic scenarios. Generally speaking, the proposed LGMDs inspired visual systems are tested in two types of roadmaps: the city ring roads, and the highway. With regard to the primary goal of this research to corroborate the LGMDs’ robustness in critical robot traffic, the roadmaps are designed with accident-prone sections resembling real world circumstances where crash often happens, e.g., the intersection challenge (Colombo and Vecchio, 2012). In addition, we also carry out comparative experiments on different densities of moving agents in the arena, and two collision sensing strategies between the bio-inspired vision and the assembled IR bumper.

**FIGURE 6:**
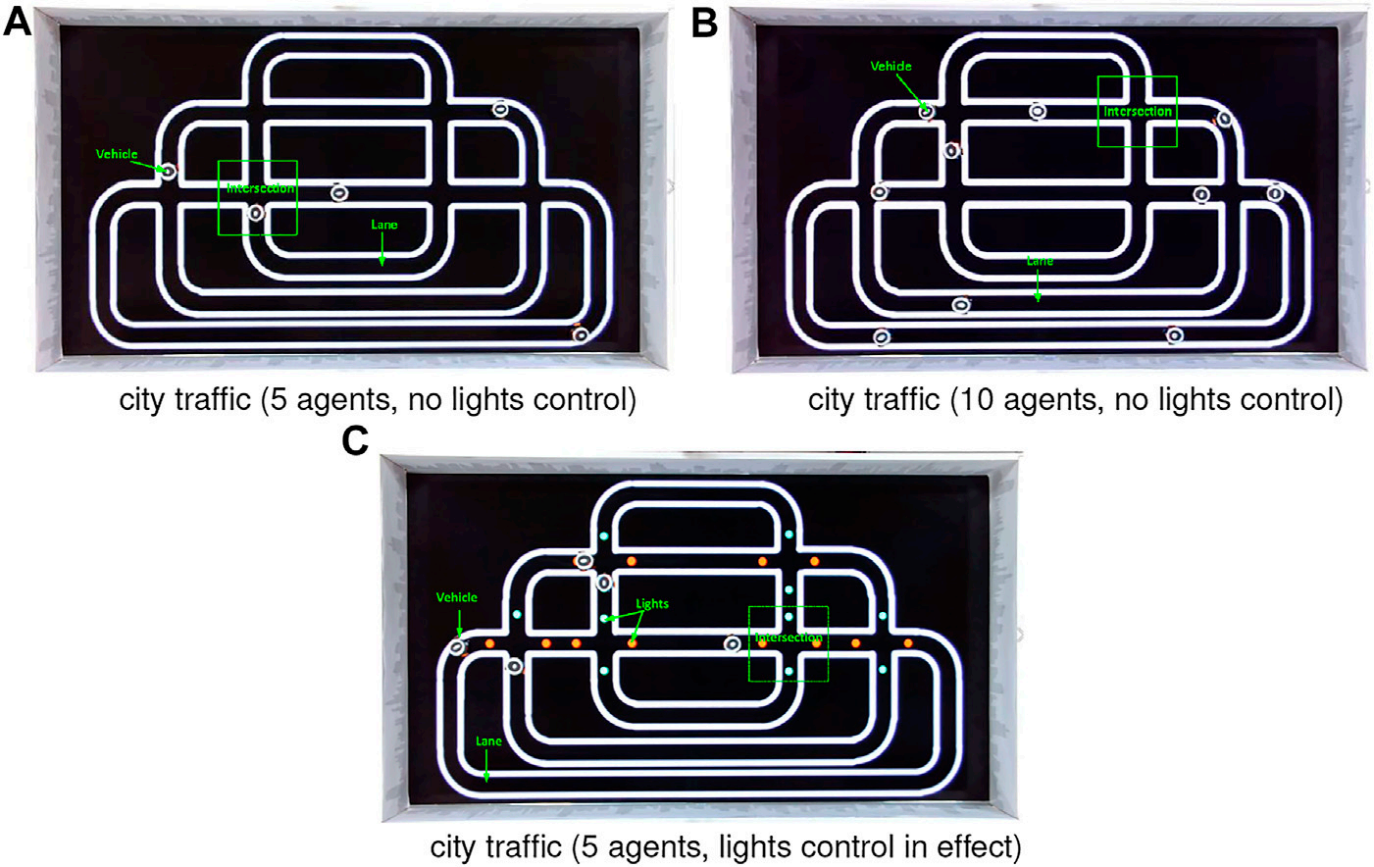
Illustration of two kinds of city ring road maps (with and without signals) from the top-down camera’s view including lanes, intersections, robot vehicles and red-green switching lights control at every crossroad. All the robots navigate unidirectionally.

**FIGURE 7:**
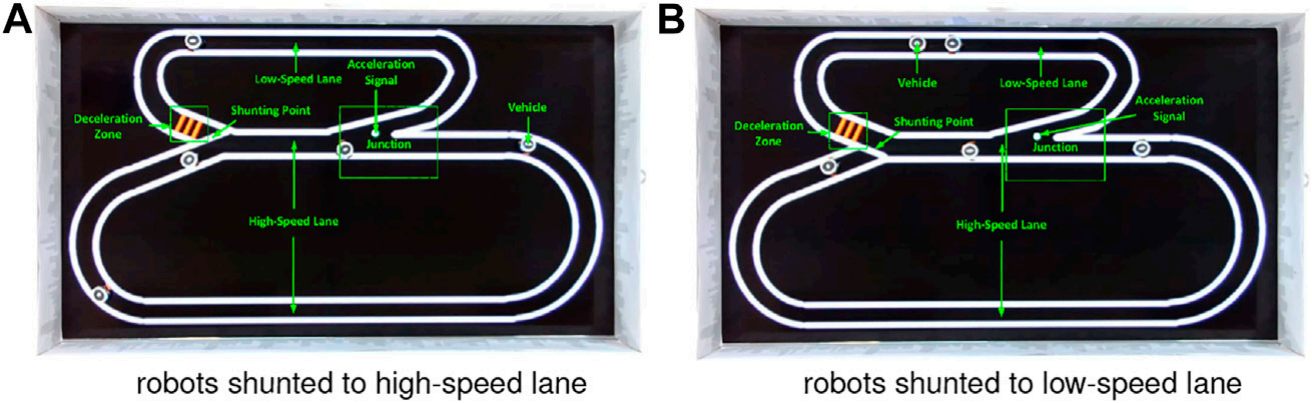
Illustration of highway traffic system including two lanes with distinct speed settings. The red signals indicate a deceleration zone as the entrance of low-speed lane. The green spot is an acceleration signal as the exit of low-speed lane. The switcher changes every 6 s. Both the two lanes are unidirectional.

**FIGURE 8:**
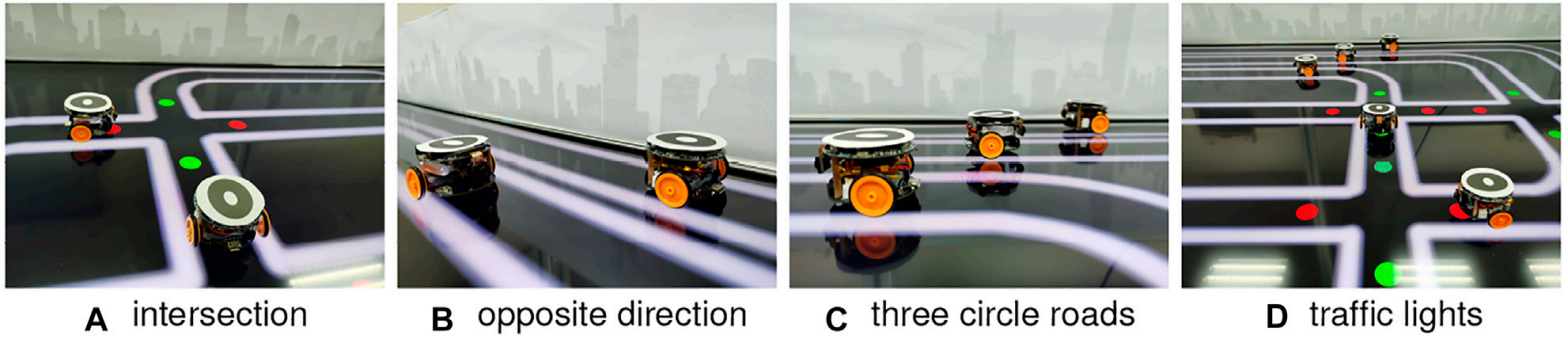
Illustration of arena inside views. The surroundings are decorated with urban skyline patterns.

Regarding the avoidance, the robot brakes abruptly once detecting potential crash and then resumes moving forward after a short break. Since we herein focus on corroborating the robustness of visual systems, the mechanical control for collision avoidance is out of the scope. Notably, the evasive behaviour matches neither the locust’s jumping/hiding, nor the many on-road situations of ground vehicles. It is also worth to emphasise that there are no human interventions in the autonomous running of multiple mobile robots unless the incidents that robot fails on route following. Each kind of robot traffic lasts for 1 hour. Figures 6, 7 and show the experimental settings from the top-down view. Figure 8 displays some arena inside views in experiments. Algorithm 1 and Algorithm 2 articulate the agent control strategies in the two kinds of traffic systems, respectively.

Here we also elucidate the relations between proposed visual systems model and control strategy. First, the model is treasured as internal component of the robot for real-time collision sensing. As presented in Algorithm 1 and Algorithm 2, the model is solely responsible for detecting potential collision; once a danger is alerted (Eq. 29), a corresponding avoidance command is sent to the motion control of robot which is prioritised over any other control logic conducted by environment. Second, the pheromone module herein is applied only to render the external “environment” for multi-robots, in order to construct roads, signals that followed, recognised by robot. Compared to other pheromone based swarm robotic studies, e.g., (Sun et al., 2019; Liu et al., 2020), the pheromones here are not released by engaged robots. More specific traffic set-ups are introduced in the following subsections.

### 3.1 Setting the City Traffic System

Firstly, the city robot traffic consists of the roadmaps without traffic lights control, and mixed by red (stop) and green (go) spot signals. Both roadmaps include straight and curving roads, and many intersections (see Figure 6). In addition, all the roads are unidirectional loops. As introduced previously, the robot navigation obeys the optical routes where the two boundaries confine its trajectory. In addition to that, the traffic lights also play roles of robot motion control. Algorithm 1 presents this kind of control logic. More concretely, if either the front or rear light sensor detects the red light, the robot will stop for a while until the light switches to green. The robot behaviour is set to go forward as default until potential crash or red light detected. The red and green lights switch every several seconds, constantly. Most importantly, all the robot agents prioritise the reaction to collision alert over traffic lights control.

In the city traffic without lights control, we also investigate the density of mobile robots at two different populations (see Figure 6). The visual scene undoubtedly becomes more complex and dynamic with more robot agents participating in the traffic system. In case of scenarios with lights control, we also set up the traffic that consists of unruly agents in half population to break the red-light control. Therefore, the intersections turn out to be the most dangerous zones resembling real-world intersection challenge (Colombo and Vecchio, 2012).

Algorithm 1: Agent Control Strategy in the City Traffic System.

1 **while** *Power on* **do**
2  Set initial forward speed randomly between 10 ∼ 14 cm/s;
3  **if** *Two-side colour sensors are between road boundaries* **then**
4   Follow route and go forward;
5   **if *Potential collision sensed* then**
6    Brake abruptly then stop for approaximately 2 s;
7   **else**
8    **If** red light detected by front or rear light sensor **then**
9     Halt the movement until green light detected;
10    **else**
11     Move forward and follow path;
12  **else**
13   Agent is derailed in collision or route following;
14   agent is manually replaced on the path
15 **end**

### 3.2 Setting the Highway Traffic System

Compared to the city traffic system that consists of many intersections as critical zones to challenge the proposed LGMDs inspired visual systems, the highway traffic system includes two lanes, i.e., low-speed and high-speed ring roads in loop, a junction where two lanes merge, a shunting mechanism to regularly separate robot vehicles into different lanes, and two light signals as the acceleration and deceleration indicators for agents, as illustrated in Figure 7. As a result, here the road junction and high speed are two leading factors of collision. Algorithm 2 presents this type of control logic. Both the two lanes are also configured as uni-directional with a shunting mechanism to separate robots with equal opportunities to follow either lanes. To change the robot’s speed, two types of signals are rendered by pheromones at the entrance and exit of low-speed lane, respectively (see Figure 7). Accordingly, the robot accelerates to enter the high-speed lane whilst decelerates preceding the low-speed lane.

Algorithm 2: Agent Control Strategy in the Highway Traffic System.

1 Navigation begins at the entrance of low-speed lane
2 Initial forward speed is randomly set between 10 ∼ 14 *cm*/*s*
3 **While** *Power on* **do**
4  **If** *Two-side colour sensors are between road boundaries* **then**
5   Follow route and go forward
6   **If** potential collision sensed **then**
7    Brake abruptly then stop for approaximately 2 s;
8     resume going forward and following route
9   **else**
__
10   **If** *Acceleration signal detected by front or rear light sensor* **then**
11    Entrance of high-speed lane reached;
12    Agent forward speed increases to around 21 *cm*/*s* within 2 s
13   **else**
__
14   **If** *Deceleration signal detected by front or rear light sensor* **then**
15    Entrance of low-speed lane reached
16    Agent forward speed decreases back to the origin within 2 s
17   **else**
__
18  **else**
19   Agent is derailed in collision or route following
20   Agent is manually replaced on the path
21 **end**

## 4 Results and Analysis

Within this section, we present the experimental results with analysis. Firstly, we demonstrate typical events of robot-to-robot interaction in the traffic systems, and visual systems output, i.e., the spike frequency of the hybrid LGMDs neural networks in the three types of investigated robot traffic scenarios. Secondly, the statistical results are given with event density maps. Lastly, we compare the proposed bio-inspired vision with another physical collision sensor in critical robot traffic. A video demo to illustrate our experiments is given in Supplementary Video.

### 4.1 Metrics

Regarding the statistical results, the overall collision avoidance rate (CAR) herein is used to evaluate the interactions between robot vehicles via the aforementioned localisation system, which is calculated by the following equations:

$$CAR\;=\;\frac{N_{ca}}{N_{E}},\;\text{where}\;N_{E}\;=\overset{T}{\underset{t=1}{\sum }}E\left(t\right),\;N_{ca}\;\;=\;\;\overset{T}{\underset{t=1}{\sum }}ca\left(t\right).$$ (30)

*E* and *ca* stand for the total robot-to-robot events and the collision avoidance with respect to time, respectively. *T* indicates the total running time of the localisation system in experiments. In this work, stop of the agent indicates a robot-to-robot event, thus:

$$E\left(t\right)\;\;=\left\{\begin{array}{cc} & 1,\;if\text{agent stops}\;\\ & 0,\;\text{otherwise} \end{array}\right.$$ (31)

With regard to the multi-robot localisation system (see Figure 3), an accomplishment of collision avoidance should satisfy the following criterion:

$$ca\left(t\right)\;\;=\;\;\left\{\begin{array}{cc} & 1,\;\text{if}\;\text{agent stops}andd_{p,q}\left(t\right)>=\gamma \\ & 0,\;\text{otherwise} \end{array}\right.$$ (32)

$$\text{where}\;d_{p,q}\left(t\right)=\sqrt{{\left(x_{p}\left(t\right)-x_{q}\left(t\right)\right)}^{2}+{\left(y_{p}\left(t\right)-y_{q}\left(t\right)\right)}^{2}}.$$ (33)

*d* is the Euclidean distance between robot *p* at position (*x*_*p*_, *y*_*p*_) and robot *q* at position (*x*_*q*_, *y*_*q*_) in the two-dimensional image plane, and *p*, *q* denote the time-varying coordinates of every two mobile robots given time *t*. *γ* = 20 (in pixels) is the predefined distance threshold to decide a successful collision avoidance in the critical robot traffic. Moreover, since the intersections and junctions are the most challenging zones for the robots that resemble the real world on-road situations, we also compare the safe passing rates (**PR**) on the intersections and junctions (*PR*_1_), as well as other road sections including the straight and curving roads (*PR*_2_). The calculations are comparable to the CAR with regional information as follows:

$$PR_{1}=\frac{N_{ca_{1}}}{N_{E}\times EPro_{1}},\;PR_{2}=\frac{N_{ca_{2}}}{N_{E}\times EPro_{2}},$$ (34)

$$\text{where}\;EPro_{1}\;\;+\;\;EPro_{2}\;\;=\;\;1,\;N_{ca_{1}}+N_{ca_{2}}\;\;=\;\;N_{ca}.$$ (35)

*EPro*_1_ and *EPro*_2_ denote the probability for critical events of interactions between engaged robots at the intersections/junctions and the other road sections, respectively.

### 4.2 Robot-to-Robot Interactions

**FIGURE 9:**
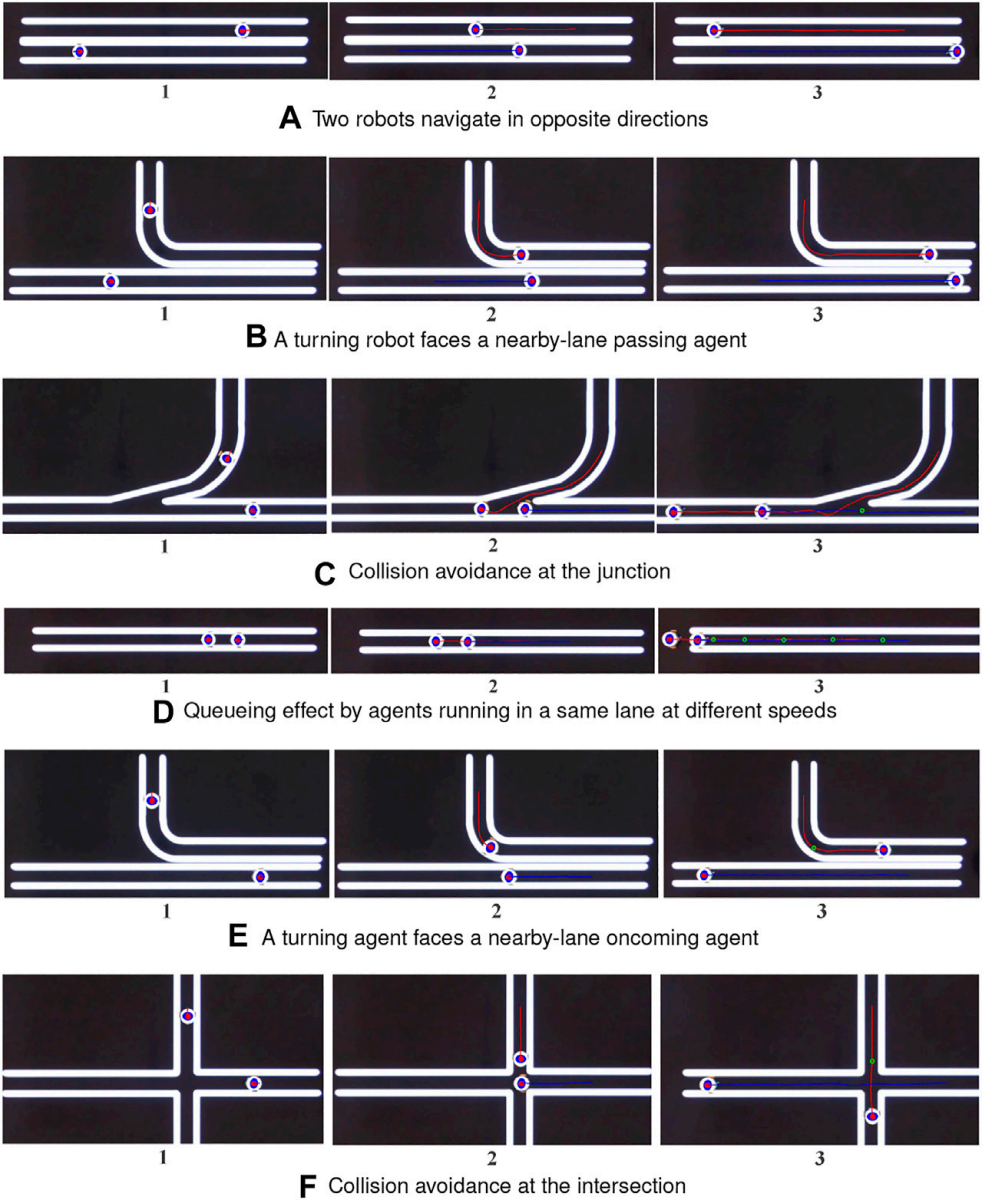
Illustrations of typical traffic phases of robot-to-robot interactions. Each phase is shown by three subsequent snapshots. The trajectories are depicted in colour lines each representing an ID-identified agent. The sites of crash avoidance are marked by green circles.

To illustrate how the autonomous micro-robots behave in the simulated traffic systems guided by the collision prediction visual systems, some typical robot-to-robot interactions are depicted in Figure 9. It appears that the avoidance behaviours are most likely aroused at some critical moments, for example, two robots meeting at the junction (see Figure 9C), queueing effect by robots on the same lane, yet at different speeds (see Figure 9D). In other normal situations (see Figures 9A,B), the robots navigate smoothly without collision avoidance. Interestingly, when the robot on curving road is facing a nearby-lane oncoming agent, there is usually an alert for a potential crash that well matches the real world driving behaviour (Sivaraman and Trivedi, 2013) (see Figure 9E). In the city traffic system, intersections are the most challenging places for robots to predict imminent crashes. When two vehicles meeting at an intersection, the urgent crossing of one agent in a very short distance could excite both the LGMDs to fire together for a positive alert (see Figure 9F). In this regard, the mimicked red-green light signals can help to alleviate the risk at intersections to a large extent like the real world on-road situations. Here we nevertheless query whether the proposed visual systems, on their own, can cope with such dangerous circumstances without the traffic signal control. The comparative experiments will be carried out in the following subsection.

### 4.3 Neural Network Response

**FIGURE 10:**
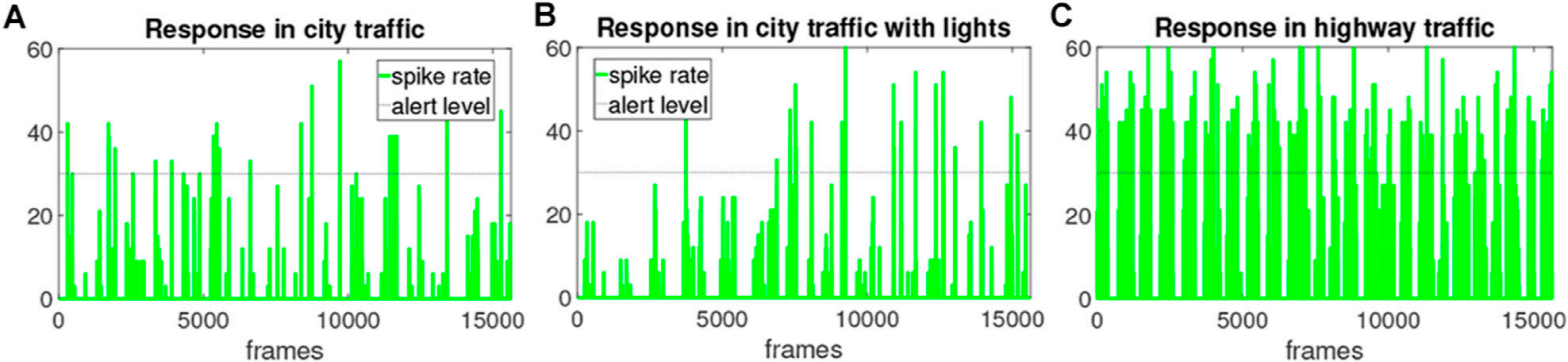
The outputs of proposed hybrid neural network model, i.e., the spike rate from a robot agent interacting within three multi-agent traffic systems, each lasting for 8 min: the horizontal dashed line indicates the alert level of firing rate.

To articulate the responses of LGMDs hybrid neural networks in different robot traffic scenes, Figure 10 illustrates three sets of model output, that is, the hybrid firing rate. Considering the introduced two types of traffic scenarios, we remotely collected the data from a robot agent interacting with others. It can bee seen from the results that a large number of collision alerts have been signalled by the LGMDs model in the embedded vision of tested robot during navigation. In addition, the neural networks respond much more constantly in the highway traffic system that consists of high-speed robot vehicles and junction.

### 4.4 Performance in Critical Robot Traffic

This subsection reports on the performance of the proposed collision prediction visual systems under constrained computation power against different robot traffic challenges. The overall CAR is given in Table 3. The comparative results on specific PR are given in Table 4.

**TABLE 3:** CAR in multi-robot traffic.

Traffic system type	Total events	Crash	CAR (%)
City traffic: No signals (5 agents)	425	81	80.94
City traffic: No signals (10 agents)	1,239	248	79.98
City traffic: Red-green lights (5 agents)	545	39	**92.84**
City traffic: Red-green lights (2/5 unruly agents)	737	129	82.50
Highway traffic (5 agents)	1,199	240	79.98

**TABLE 4:** PR in multi-robot traffic.

Intersection and junction: EPro_1_ and PR_1_, other sections: EPro_2_ and PR_2_				
Traffic system type	EPro_1_ (%)	PR_1_ (%)	EPro_2_ (%)	PR_2_ (%)
City traffic: No signals (5 agents)	53.65	71.49	46.35	91.88
City traffic: No signals (10 agents)	55.21	73.54	44.79	87.93
City traffic: Traffic lights (5 agents)	**60.0**	**91.74**	40.0	**94.50**
City traffic: Traffic lights (2 unruly in 5)	59.43	77.85	40.57	89.30
Highway traffic (5 agents)	43.20	75.29	**56.80**	83.55

#### 4.4.1 Performance in the City Traffic System

**FIGURE 11:**
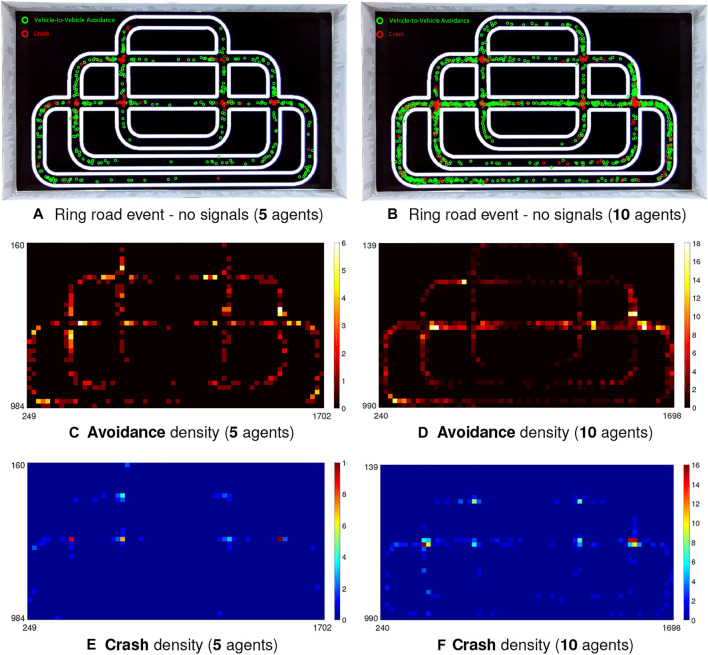
Illustration of event and density maps from top-down view of camera including avoidance and crash in the city traffic system at two comparative populations of robot vehicles without light signals at intersections. **(A, B)** Event maps: red and green circles indicate the positions of crash and avoidance events between robot vehicles, respectively. **(C–F)** Density maps: X-Y plane denotes the image coordinates.

In the city traffic system, we carry out systematic and comparative experiments involving several cases. In the first case, the robot traffic has no signal controls at intersections. We also look deeper into the density effect on collision prediction performance. Figure 11 illustrates the event and density maps of all micro-robot agents engaging in the ring road traffic for 1-h implementation.

**FIGURE 12:**
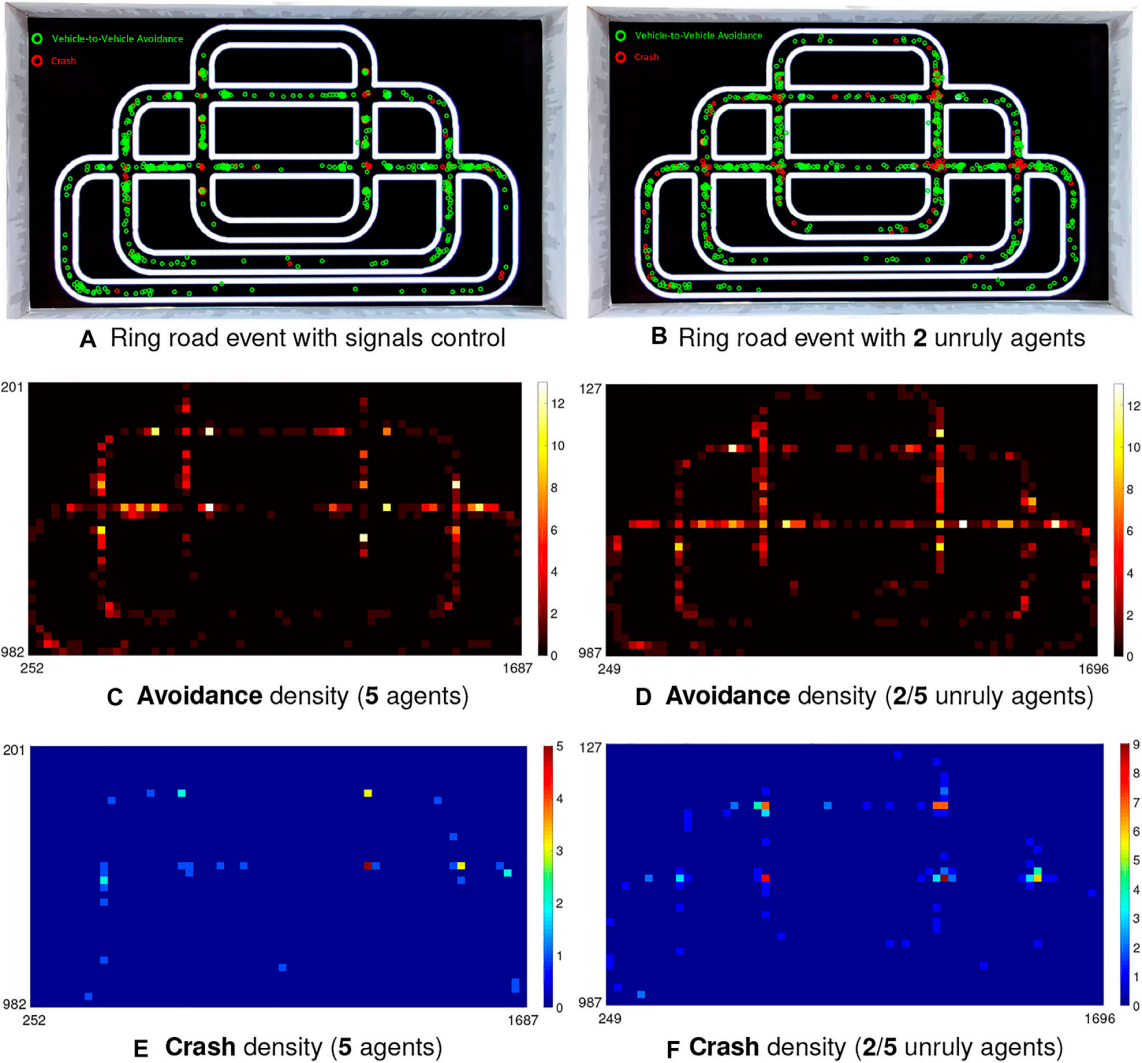
Illustration of event and density maps in the city traffic system (5 robot vehicles) with signals control and engaged unruly agents at intersections.

In the second case, the red and green switching traffic lights are used as the auxiliary signals for robot flows control at intersections. An interesting episode is plotted in this case by mixing unruly robot agents not obeying the law of traffic signals, i.e., red to stop and green to go, that mimic the drivers who always break the traffic rules at intersections leading to immense on-road safety issue. Figure 12 illustrates the corresponding results at this point.

Together with the statistical results in Tables 3, T4, we have the following observations on the experiments of city traffic system:

1) Table 4 shows that more than half critical events take place at intersections in all the imitations of city ring road traffic (see EPro_1_ in Table 4) indicating that our robot traffic could reflect real world road challenges (Colombo and Vecchio, 2012).
2) Compared to the performance at intersections, the PR is quite higher in the straight and curving road (all above 80%). To be more intuitive, Figures 11, 12 also demonstrate that the crash most frequently occurs at intersections with relatively lower PR, which show higher crash densities there; on the other hand, the PR is fairly higher in other road sections corresponding to higher avoidance densities.
3) The overall CAR peaks in case of the city traffic system with lights, and without unruly agents (92.84%). Compared to that, the CAR reaches valley once lacking red-green signals to relieve the traffic flows at intersections (80.94%).
4) On the aspect of density comparison, there is merely tiny difference on both the CAR and PR of two investigated populations, which reveal that the proposed visual systems perform robustly for collision prediction even in more dynamic environment.

Generally speaking, the proposed bio-inspired hybrid neural networks work effectively and consistently on collision prediction in the city traffic system despite that the intersections are still posing challenges on timely detection-and-avoidance using the visual approach as the only modality. However, we believe this can be improved by increasing the view angle of optical sensor as the current view of frontal camera can only reach approximately 70°. The risk of intersection could also be alleviated by sensor fusion strategy, or other algorithms in intelligent transportation system (Colombo and Vecchio, 2012). With discrepancies amongst forward velocities of multi-robots (refer to the setting in Algorithm 1), the robot vehicles well demonstrate queueing effect guided by the collision prediction systems. On the straight and curving roads, the LGMDs inspired visual systems perform more robustly and consistently on collision alert in comparison with the intersections. Additionally, the robot density in the traffic system dose not greatly affect the overall performance of visual systems, which imply the proposed bio-inspired computation is robust and flexible to more dynamic visual scenes.

#### 4.4.2 Performance in the Highway Traffic System

**FIGURE 13:**
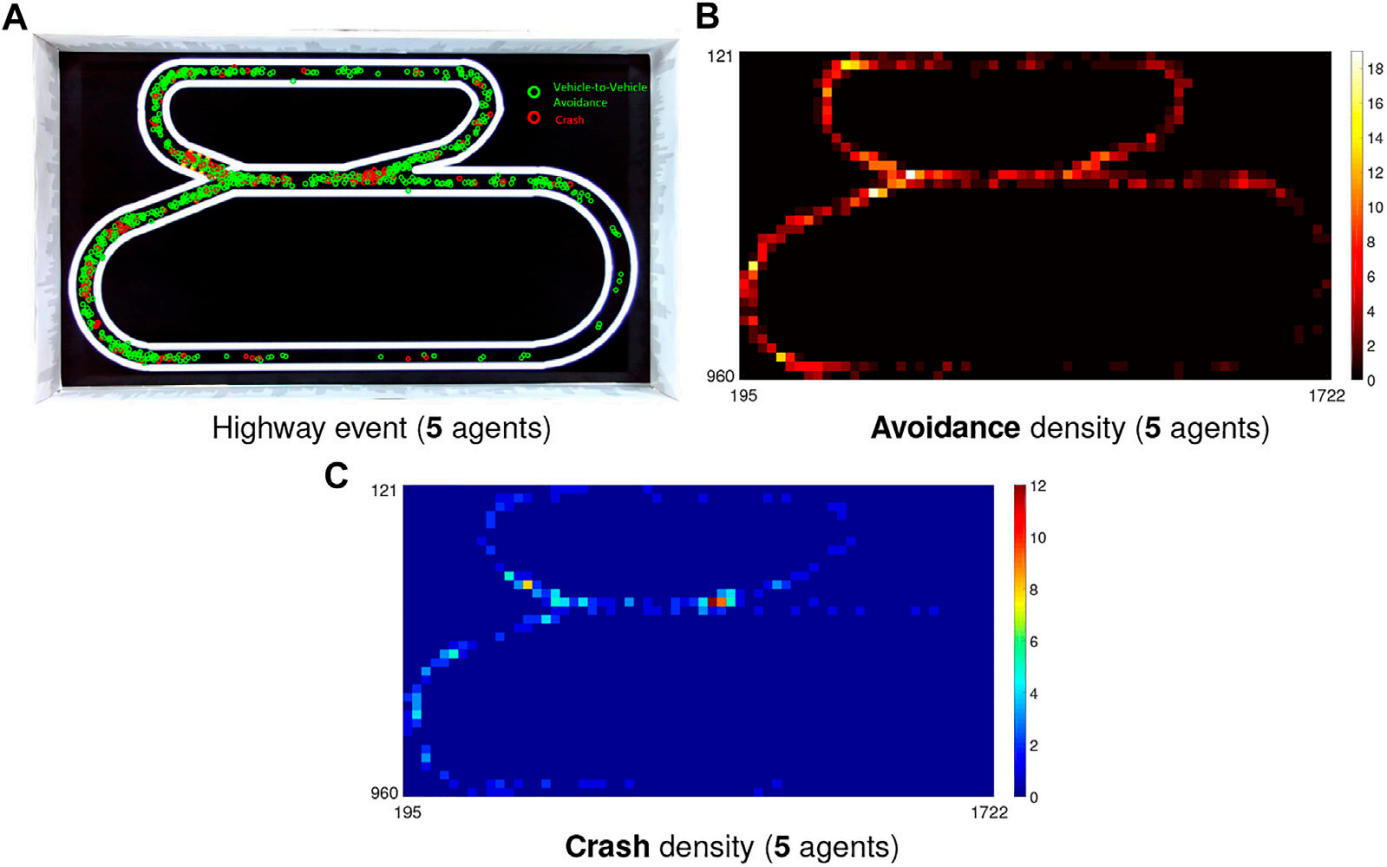
Illustration of event and density maps in the highway traffic system.

In the critical highway traffic system, two lanes separate the speed of robots into two ranges, as presented in Section 3. The overall CAR and PR are given in Tables 3, T4. In addition, Figure 13 illustrates the results with event and density maps. Here the most noticeable observation is that in comparison with the city traffic system barring the 10-agents case, more than twofold critical events take place in the highway traffic within the 1-h implementation. Table 4 clearly shows that nearly half (43.2%) critical events concentrate at the junction where the high-speed and low-speed robot flows merge. In spite of that, the overall CAR remains fairly high that is in consistent with the city traffic system without signals control (79.98%); the PR at either the junction (75.29%) or the other road sections (83.55%) is slightly lower than the city traffic results. In general, the LGMDs inspired visual systems are robust to cope with collision prediction in high-speed, dynamic visual scene, in the micro-robot under constrained computation cost.

On the other hand, we also find challenges through the experiments. The event density maps in Figure 13 demonstrate that it is still difficult to address the crash avoidance problems at the junction where the low-speed robot vehicles are accelerating to merge into the high-speed flow. At this point, the robots are required to form a queue to pass the junction free of collision. The similar situations happen at the deceleration zone where the high-speed vehicles are shunted to queue into the low-speed flow. In addition to that, compared to the city traffic results, the PR in other sections is relatively lower, i.e., more crashes between robots occur on the high-speed curving road (see Figure 13C).

### 4.5 Sensor Comparison

Through the previous experiments, we have shown the effectiveness and robustness of LGMDs inspired visual systems for timely collision prediction in critical robot traffic. The energy efficiency have also been verified via the successful implementation on the micro-robot under extremely constrained computation power. As an alternative, optical approach to collision detection, the proposed bio-inspired computation could be scalable across various platforms and scenarios. In the last type of experiments, we also compare this visual approach with another classic physical sensor strategy–the IR bumper sensors used extensively in robotics for collision sensing and avoidance.

**FIGURE 14:**
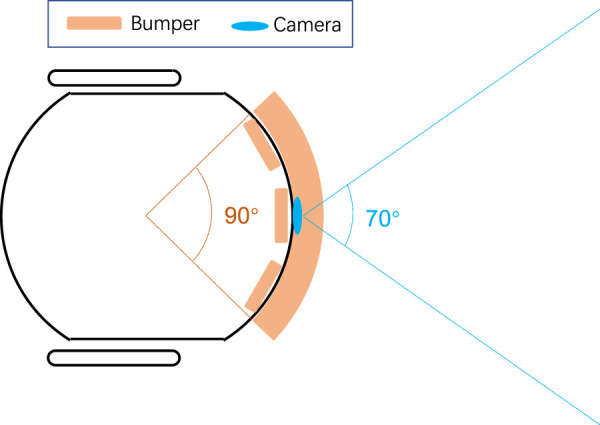
Schematic comparison on sensing range of two physical sensor strategies between the combination of three IR bumper sensors and the frontal monocular camera.

The micro-robot possesses three IR sensors as short-range obstacle sensing technique. Figure 14 compares the detection range between the two physical sensor strategies. It appears that the combination of three bumper sensors has wider coverage in space up to approximately 90° than the monocular camera which could reach only 70°. On the other hand, the optical sensor has much longer sensing distance with respect to the advantage of optical methods. In this kind of experiments, the robot vehicle applies the same braking avoidance behaviour guided by the bumpers. The other experimental setting is in accordance with the earlier experiments. Each type of traffic system implementation lasts for 1 hour, the same duration.

Table 5 lists the CAR of IR based technique in the two traffic systems. Though with wider coverage in front, here the CAR of IR based technique is much lower in both types of traffic system (66.81% in city traffic without lights and 48.30% in highway), compared with the performance of LGMDs inspired visual systems (80.94% in city traffic without lights and 79.98% in highway). Compared to the proposed optical approach, the short-range technique can not fulfil the timely crash prediction in the critical robot traffic. The short distance between interactive robots brings about a smaller amount of critical events within an identical time window. Besides that, the CAR is even lower in the highway scenario, which points out that the IR short-range detector is unsuitable to predicting high-speed approaching objects very timely; whilst the proposed approach can signal an impending crash quite earlier. With more abundant features extracted, filtered from the dynamic visual scene, the hybrid LGMDs inspired visual systems are more robust in collision prediction.

**TABLE 5:** CAR of IR sensors in multi-robot traffic.

Traffic system type	Total events	Avoidance	CAR (%)
City traffic: No signals (5 agents)	226	151	66.81
Highway traffic (5 agents)	176	85	48.30

## 5 Discussion

In this section, we discuss further on observed problems through the experiments, and point out corresponding future works. Firstly, we have seen some limitations of the proposed approach for quick collision detection in the context of robot traffic. Some critical conditions are still challenging the proposed LGMDs inspired visual systems. In the city traffic system particularly without signals control, crashes generally take place at intersections (see Figure 11). During the experiments, we have observed that there is a possibility that two robot vehicles are arriving at the intersection, simultaneously. The current approach as frontal collision sensing technique can not well cope with such a problem. On the other hand, crashes are significantly reduced if the robots reached intersection in succession, such as the example shown in Figure 9F; the successful avoidance density is fairly high near the intersections in the city traffic system, as shown in Figures 11, 12. The proposed approach can predict a danger by nearby object crossing the field of vision, very robustly and timely. In this research, a possible restriction is the limited view angle of the monocular camera system in the micro-robot. Therefore, we will develop binocular and panoramic camera systems for future scientific study.

In the highway traffic system, we have noticed that the very high speed movement, i.e., robot velocity over 20 cm/s in the context, is another problem to the LGMDs inspired visual systems embedded in the micro-robot vision. In our previous studies on LGMD-1 (Fu et al., 2018b) and LGMD-2 (Fu et al., 2019b), we have figured out that the LGMDs models demonstrate speed response to approaching object, i.e., the neural networks deliver stronger output against faster approaching object at higher angular velocity. The speed response and looming selectivity of LGMDs models is achieved by the competition between excitation and two kinds of inhibitions–the lateral inhibition, and the FFI. Most importantly, the former inhibition works effectively to sculpt such selectivity when objects expanding on the field of vision before reaching a particular angular size. Otherwise, the FFI (or PM in the LGMD-2) mechanisms could immediately suppress the LGMDs at some critical moments like the end of approaching, the start of receding. Accordingly, the proposed visual systems in the high-speed moving robot have always confronted such difficult situation. This gives reasonable explanation on the higher crash density near the junction where the two lanes merge (see Figure 13). In another word, the high-speed agent could not appropriately predict a crash with the emerged low-speed agent at the junction. In this regard, future effort is in demand to enable the visual systems to well cope with ultra-fast approaching movements.

Regarding the control strategy, as it is not the focus of this research, we have applied very basic switch control between two states, i.e., move and stop, in order to enable the robots to tightly follow paths in all kinds of traffic systems. As a result, the potential crash avoidance is led by abrupt braking which can not fulfil the very complicated, real world emergency actions of vehicles. For example, the deceleration earlier to urgent stop has not been involved in the control of micro-robots. We will incorporate in the robotic motion system more advanced control method, e.g., the fuzzy control, to enrich the robot avoidance reaction corresponding to more realistic behaviours (Sivaraman and Trivedi, 2013).

Secondly, in comparison with previous robot arena experiments on the LGMDs inspired visual systems, in which the robot motion was not confined by specific trajectories (Fu et al., 2017; Fu et al., 2019b), this study strictly binds the robot motion in navigation (see Figure 5, Algorithms 1, 2). The prioritised goal of robot motion is to tightly follow the paths desired by the robot traffic implementation. However, the current motion strategy has the flaw that the robots usually experience yaw rotations in route following. This sometimes results in false positives of collision alert. We will explore new methods in the LGMDs neuronal system model to habituate such visual movements, and also improve the robot route following strategy.

Last but not least, the robot vehicles currently are not fully autonomous in traffic systems. Despite human interventions in merely specific conditions during experiments (e.g., the robot fails in route following or collide with other agents), the human-robot interactions have still influenced the robot traffic implementation, e.g., manually replacing the robot on routes after crash. Accordingly, the different robot traffic systems need to be verified with respect to (Fisher et al., 2021). The safety and functional correctness of the robot traffic reflecting some real world scenes also need to be further validated according to (Webster et al., 2020).

## 6 Concluding Remarks

This paper has presented a novel study on investigating bio-inspired computation approach to collision prediction in dynamic robot traffic reflecting some real world on-road challenges. To fill the scientific study gap on evaluating online artificial visual systems in dangerous scenarios where physical crashes are prone to happen, we have applied a recently published robotic platform to construct traffic systems including the city roadmap with many intersections, and the highway with junctions. To sharpen up the acuity of collision detection visual systems to darker objects approaching over other categories of movements like recession, we have integrated two LGMDs neuronal models, i.e., the LGMD-1 and LGMD-2 neural networks, as a hybrid model outputting alert firing rate. A potential collision is predicted only when both the LGMDs are highly activated. To focus on investigating the proposed collision prediction visual systems, we have applied the simple bang-bang control to allow the robot to tightly follow paths and brake abruptly corresponding to the avoidance action. The arena experiments have verified the robustness of the proposed approach to timely collision alert for engaged robot vehicles in the traffic systems. This research has complemented the previous experimentation on such bio-inspired visual systems in more critical real-physical scenarios, under extremely constrained computation power. This also has provided an alternative, energy-efficient technique to current collision alert systems. The propose visual systems can be transformed into neuromorphic sensing paradigms which could be prevalent for future autonomous machines.

## Data Availability

All datasets generated for this study are included in the article/Supplementary Video.
